# Shared decision-making endorses intention to follow through treatment or vaccination recommendations: a multi-method survey study among older adults

**DOI:** 10.1186/s12911-024-02611-2

**Published:** 2024-07-23

**Authors:** Tuuli Turja, Milla Rosenlund, Virpi Jylhä, Hanna Kuusisto

**Affiliations:** 1 Faculty of Social Sciences, Tampere University, Kalevantie 5, Tampere, 33014 Finland; 2 Faculty of Social Sciences and Business Studies, Department of Health and Social Management, University of Eastern Finland, Yliopistonranta 1 F, Kuopio, 70211 Finland; 3 Department of Neurology, Tampere University Hospital, Tampere, Finland; 4 Wellbeing Services County of North Savo, Research Centre for Nursing Science and Social and Health Management, Kuopio University Hospital, Puijonlaaksontie 2, Kuopio, 70211 Finland

**Keywords:** Adherence, Clinical encounters, Multi-method survey, Patient involvement, Shared decision-making

## Abstract

**Background:**

Previous studies have shown that shared decision-making (SDM) between a practitioner and a patient strengthens the ideal of treatment adherence. This study employed a multi-method approach to SDM in healthcare to reinforce the theoretical and methodological grounds of this argument. As the study design, self-reported survey items and experimental vignettes were combined in one electronic questionnaire. This technique aimed to analyze the effects of previous experiences and the current preferences regarding SDM on the intentions to follow-through with the medical recommendations.

**Method:**

Using quantitative data collected from the members of the Finnish Pensioners’ Federation (*N* = 1610), this study focused on the important and growing population of older adults as healthcare consumers. Illustrated vignettes were used in the evaluation of expected adherence to both vaccination and the treatment of an illness, depending on the decision-making style varying among the repeated scenarios. In a within-subjects study design, each study subject acted as their own control.

**Results:**

The findings demonstrated that SDM correlates with expected adherence to a treatment and vaccination. Both the retrospective experiences and prospective aspirations of SDM in clinical encounters supported the patients’ expected adherence to vaccination and treatment while decreasing the probability of pseudo-compliance. The association between SDM and expected adherence was not affected by the perceived health of the respondents. However, the associations among the expected adherence and decision-making styles were found to differ between the treatment and vaccination scenarios.

**Conclusions:**

SDM enables expected treatment adherence among older adults. Thus, the multi-method study emphasizes the importance of SDM in various healthcare encounters. The findings further imply that SDM research benefits from questionnaires combining self-report methods and experimental study designs. Further cross-validation studies using various types of written and illustrated scenarios are encouraged.

**Supplementary Information:**

The online version contains supplementary material available at 10.1186/s12911-024-02611-2.

## Introduction

Patient autonomy, empowerment, and individually applied involvement in decision-making are cornerstones of high-quality healthcare services [1–3]. Shared decision-making (SDM) is a process in planned decision-making that involves both the patient and healthcare professional. Thus, successful SDM relies on applying a combination of high-quality medical evidence with information provided by the patients on their personal values and preferences [4]. SDM is especially used in so-called “preference-sensitive” conditions in which there are multiple treatments with similar efficacy but different safety profiles, follow-up, and administrations [5]. There is a rapidly increasing amount of evidence in various specialties that patients consciously prefer SDM, and that patients involved in SDM experience less decisional conflict and regret, as well as increased knowledge, satisfaction, adherence, and, ultimately, better health outcomes [6, 7]. Older adults particularly, are known to appreciate SDM in healthcare [8–10].

This study investigated decision-making in clinical encounters to reinforce the theoretical and methodological grounds of SDM endorsing the follow-through of medical recommendations. The study used a sample of Finnish pensioners as the exemplary population using general healthcare services. Finland is of particular interest because of the reform of public healthcare, social welfare, and rescue services in 2023. Finland is also among the five fastest-aging populations in the world [11]. The share of citizens aged 65 and older was 15% in 2000, 22% in 2021, and is expected to reach nearly 25% by 2070 [12]. Consequently, the aging population challenges societies in many ways as older people with multimorbidity become more prevalent users of health and social services [13, 14]. The goal of the forthcoming health and social services reform in Finland was to guarantee equality and quality in health and social care services in a situation in which the population is aging and the share of the working population is declining [15]. The reform also included outlooks for digitizing health and social services that, in part, emphasize the ideologies of active citizens who are motivated and capable of participating in the decision-making regarding the services they receive [16].

## Background

Medical decision-making includes styles such as authoritarian, in which decisions are made solely by healthcare professionals; guided decision-making, in which professionals share information but reserve the final conclusion for themselves; simple decision-making, in which the decision is left to the patient; and SDM, in which options, risks, benefits, values, and preferences are discussed and agreed upon between the practitioner and the patient in consensus. Among the various decision-making styles, the simple decision-making style is viewed as a representation of modern consumerism prioritizing patient autonomy [17]. SDM, on the other hand, prioritizes greater patient involvement and person-centeredness with its objective to utilize both clinical and personal information to make the best decisions in the individual cases [18, 19].

SDM seeks to build the shared understanding of care needs and interventions that will be effective, relevant, acceptable, and meaningful in individually and contextually varying situations. The major benefits of SDM are the increase of interaction between the patient and the practitioner, and how the patient has an emphasized opportunity to share information they consider important and relevant. In SDM, treatment options are carefully explained by healthcare professionals and jointly agreed upon with the patients, who will receive information about the risks and benefits underlying each option [7]. A systematic review of randomized controlled trials has shown that SDM can be an effective method for reaching a treatment agreement [5]. Specifically, SDM is found to improve affective-cognitive outcomes, such as improved satisfaction and less decisional conflict after clinical encounters, all of which will have a positive effect on well-being of the patients [7]. Furthermore, SDM interventions may improve the health outcomes of disadvantaged groups more than those of more proactive patients with higher information literacy [5]. That said, the use of SDM itself entails the opportunity to increase knowledge, understanding and self-efficacy among patients, to promote informed choice and participation in decision-making, and to reduce decisional discord during and after clinical encounters [20, 21].

Older people perceive great value in SDM and mutual trust in their relationships with clinicians [8, 10, 22]. SDM is particularly associated with a positive power balance between the clinician and the patient, as well as the ideal way information is provided [23, p. 4]. Many times, patients may feel that they are not informed at a sufficient level or are unable to participate in decision-making as much as they would like to. This situation can create the risk of a patient leaving an appointment feeling misunderstood or disregarded [24].

A recent study has shown that the clinicians’ perception of medicolegal vulnerability is a barrier to the implementation of SDM [9]. This includes the expectations to follow clinical guidelines and to apply them regardless of difficulties, as well as to communicate clinical uncertainty also as a part of SDM. The medicolegal aspects refer specifically to the clinicians’ concerns about being vulnerable to professional or legal challenges in the event of negative outcomes. As another barrier, patients and doctors in primary care consider the time for consultation to be too limited and that there exists a distinct need for building continuity into the doctor-patient relationship [9]. It is implied that, for people to participate in SDM they require support, knowledge, and clearly and individually communicated information regarding their conditions and care [25, 26].

As an outcome of doctor-patient interaction, adherence to treatment form a priority for the reason adherence is considered as a direct predictor of the patient’s health [27, 28]. Shared information alone has been acknowledged as a significant factor in treatment and vaccination recommendation adherence [29–31]. In a similar vein, participatory actions, in general, are means to effectively reduce failure demand and other negative outcomes in healthcare [32, p. 3]. Failure demand caused by (a perception of) unsuccessful service [33] is a prevalent phenomenon in primary care, particularly among older patients [34].

Another negative outcome in healthcare encounters is pseudo-compliance, in which the patient apparently agrees with the practitioner’s advice but exits the appointment with no intention to follow the recommendations or the care plan [35, p. 138]. In a study of adult patients in an emergency ward, 16% of the patients were found to exhibit pseudo-compliance [36]. Existing research literature has not yet answered the question regarding whether pseudo-compliance is a relevant risk in situations of SDM or if it is associated only with less participatory styles of decision-making in clinical encounters. There are implications that pseudo-compliance is more common among individuals with lower levels of health literacy [36]. Pseudo-compliance is a type of nonadherence that can be either intentional or unintentional [37]. Intentional nonadherence is associated with, for example, motivation and patients’ resistance to taking medications. Conversely, unintentional nonadherence is related to patients’ skills and abilities [38]. Again, health-literate individuals are found to be at less risk of exhibiting unintentional nonadherence to treatment or medication [39, 40].

This study focused on older adults as an important but generally overlooked group of healthcare service users. Medical decision-making was examined through previous patient experiences and current preferences using a multi-method questionnaire that combined retrospective survey items (SDM-Q-9) and prospective vignettes (illustrated scenarios from a clinical appointment) [41]. In the objective to study SDM in healthcare encounters, the first task was to assess the novel, experimental methodology introduced in the questionnaire, and the second task was to use the multi-method survey data in analyzing the association between SDM and the patients’ intentions to follow through with medical recommendations.

First, comparing the self-designed SDM vignettes to the standardized SDM scale, we examined *the cross-validity of experimental vignettes in studying the decision-making styles in clinical encounters*. The vignette-testing hypothesis was that prior experiences and present motivation toward SDM correlate positively with each other (H1). The assumption of compatibility between prior and present views on SDM underlie the premise of the views toward SDM remaining relatively sustained over time and over a range of clinical encounters [42].

Second, using a multi-method approach to SDM, the association between decision-making and expected treatment adherence was examined with the specific research question: *How can decision-making processes in clinical encounters affect patients’ intentions to follow through with treatment or vaccination recommendations* (hereafter referred to by the more concise term ‘expected treatment/vaccination adherence’). The variation in the expected adherence was presumed to be associated with the theoretically relevant predictors of SDM contained both in retrospective SDM measures and in prospective vignettes. In the second set of hypotheses, the assumption was that prior experiences, together with present motivations toward SDM, associate with expected adherence to vaccination (H2a) and treatment (H2b).

## Method

This study used electronic questionnaire data collected from the members of the Finnish Pensioners’ Federation (FPF) from December 2021–January 2022. The invitation to participate was sent by the FPF to their 120,000 members whose e-mail addresses were registered in the member database (*N* = 30,329). The response rate of 5.3% (1.34% of the population) resulted in a sample of 1,610 respondents (M_age_ = 70.9, SD_age_ = 5.67, 60.6% female). The respondents reported using healthcare services regularly, the mode in the frequency being *several times per year* (70.3% of the responses). A fifth of the respondents (19.6%) reported using healthcare services *once per year or less*, 9.1% reported *monthly* use, and 1% reported *weekly* use. The respondents were also asked about their subjective health with the question: “How do you perceive your current health status?” used widely in questionnaires and presented identically in the national FinSote survey [43]. The distribution of the responses was as follows: “good” 22.1%, “fairly good” 43.7%, “average” 28.0%, “fairly poor” 6.1%, and “poor” 0.1%.

The questionnaire was developed for this study in Finnish language. The translation to English is available as a supplementary file. The questionnaire consisted of two sections. The first section included direct multiple-choice items about the respondents’ demographics, other background information, and retrospective questions about the participants’ *experiences* of SDM as they related to their treatment and care. In the second section, respondents were introduced to an experimental vignette study that was used to measure their current *preferences* to involve themselves in the decision-making related to their treatment.

The retrospective views about prior experiences of SDM were measured using the widely utilized and validated SDM-Q-9 scale [44, 45]. SDM-Q-9 is a brief self-report scale measuring the patients’ subjective views on their level of involvement in decision-making in healthcare encounters. SDM-Q-9 does not measure a patient’s desired involvement in decision-making, per se, although this interpretation is sometimes mentioned in the discourse surrounding the scale. The task of the respondents was to evaluate their previously realized level of decision-making in a clinical appointment. Most of the respondents had received a diagnosis at the latest appointment (44%) or the visit had concerned a chronic condition (41%).

The respondents who reported having met with a doctor within a 6-week time frame were taken to the SDM-Q-9 scale on the questionnaire. These particular respondents (*n* = 629) were asked to recall their last appointment and give their responses on a scale from “totally disagree” (1) to “totally agree” (4). The descriptive statistics for the SDM-Q-9 items are shown in Table 1. Retrospective self-report studies are especially challenging among the samples of older people [46]. However, optimizing the time frame of remembrance between a month and a year can make a significant difference [47]. A six-week time frame has been found to produce accurate retrospective information about healthcare encounters among older patients [48].

**Table 1:** Descriptives for SDM-Q-9 scale (*N* = 629)

Item	Mean	SD	α
1 The doctor made it clear that a decision needs to be made.	3.40	0.88	
2 The doctor wanted to know exactly how I want to be involved in making the decision.	2.85	1.06	
3 The doctor told me that there are different options for treating my medical condition.	2.83	1.09	
4 The doctor precisely explained carefully the advantages and disadvantages of the treatment options.	2.79	1.11	
5 The doctor helped me to understand all the given information.	3.23	0.94	
6 The doctor asked me which treatment option I prefer.	2.47	1.20	
7 The doctor and I thoroughly weighed the various different treatment options.	2.57	1.19	
8 The doctor and I selected a treatment option together.	2.94	1.16	
9 The doctor and I reached an agreement on how to proceed.	3.31	0.99	
TOTAL	2.93	0.32	0.92

### Vignettes

Instead of applying a control preference questionnaire to allow participants to assess their preferences and motivations for involvement in decisions about their treatment, we chose an experimental, projective study design using illustrated vignettes [49, 50]. Applying two distinct approaches, in this case, explicit and experimental methods, is an effective way to minimize common method bias in surveys. Because of the well-established and standardized SDM-Q-9 scale, the vignette section of the survey was introduced and assessed as an additional novel methodology. The vignettes presenting scenarios from a clinical appointment were assumed to work as ambiguous stimuli to the respondents instead of collecting conscious evaluations of the situation. The additional value of the multimethod questionnaire lies in the aim to decrease the response biases plaguing the explicit self-report methods [51, 52].

In this experimental vignette setting, as the second section of the survey, the “paper people method” was utilized [41]. This type of method has been used widely in ethical decision-making studies in which it is difficult to study sensitive topics in an experimentally controlled setting. The respondents were asked to relate to the repeated illustrated scenarios in the role of the patient (Appendix A). The scenarios presented interactions between a doctor and a patient in which the decision-making style varied in four conditions: authoritarian, guided, simple, and SDM [53]. The scenarios were presented in written form and were accompanied by illustrations that highlighted key aspects of the decision-making process being studied. To make the scenarios as comprehensible as possible, the variation in the illustrations were also presented as symbols. All of the situations made use of the same symbols. The mental accessibility of the scenarios is understood to be improved by the inclusion of pictures and symbols [54].

After each scenario ended in an apparent consensus between the practitioner and the patient, the respondent was asked about their intention regarding adherence to treatment within the scenario. Thus, the prospective vignette study was used to measure peoples’ motivation to involve themselves in the decision-making related to their treatment. After the scenarios, the responses referring to intentional non-adherence were indicated pseudo-compliance. In order to rank the four conditions of decision-making according to the respondents’ preferences, Friedman’s ANOVA with “best-worst” scaling was used as an analysis method. The best-worst method is widely used in person-centered study designs [55], however, primarily for gathering information for clinical decisions, not for studying decision-making preferences [56, 57]. Diverging from the typical use of best-worst scaling, but consistent with traditions in experimental study design, the current study did not reveal to the respondents the preference ranking between the four decision-making conditions. The methodological strategy was to retain the variation among the vignettes concealed by introducing the parallel scenarios as individual cases to be evaluated on the basis of adherence to a treatment recommendation. In other words, the respondents were not tasked with scaling the different options from best to worst; instead, the ranking was drawn from the responses.

### Statistical testing

After preliminary univariate analysis, multivariable analyses were conducted to test the hypotheses. Binary logistic regression models are presented separately for vaccination and treatment scenarios, and the results are reported by regression coefficients (B), odds ratios (OR), and coefficients of determination (pseudo-R^2^). In logistic regression, pseudo-R^2^ indicates model fit between parallel models instead of giving generalized information about the explanatory power of the model.

Before the actual data collection, the questionnaire was piloted for the face validity of the items. The pilot data included a separate convenient sample of total of nine patients of different sociodemographic profile who had lately visited doctor’s appointment. Majority of them used health care services a few times per year. Respondents evaluated the importance and comprehensibility of the survey items. On separate sections in the questionnaire, they had the possibility to give open-ended remarks about the survey. The items were modified according to the feedback for their phrasing and internal order.

## Results

### SDM scale

The aggregate scale of SDM-Q-9 (Table 1) ranged from 9 to 36 (M = 26.39, SD = 7.56). The realization of SDM in the previous clinical appointment was slightly more probable among the respondents who reported better general health (*r* = 0.16, *p* < 0.001).

The retrospective views in the SDM scale correlated with the prospective views of decision-making. The correlations were weak throughout the variables (*r* = 0.10–0.21, *p* < 0.001). This shows the distinct constructions in which SDM is understood as the *experience* in the involvement of decision-making, and the vignette portion of the study measured the motivational *preferences* between the decision-making styles. Regarding adherence to a vaccination recommendation, the highest codirectional correlation was found between the experienced SDM and the preference of SDM (*r* = 0.13, *p* < 0.001). Regarding adherence to a treatment recommendation, the highest correlation was found between the experienced SDM and the preference of guided decision-making (*r* = 0.21, *p* < 0.001).

### Vignette study

Friedman’s ANOVA on Ranked Data demonstrated consistency in the evaluations of the preferred styles of decision-making. SDM was associated with adherence in both vaccination and treatment scenarios (Table 2). Because of the relatively large sample size, the significance is interpreted from the effect size of Kendall’s *W.* Small effect sizes of 0.02 (vaccination) and 0.03 (treatment) imply only a marginal consistency in the differences between the decision-making styles. Nevertheless, Friedman’s test rejecting the null hypothesis gives sufficient reason to conclude SDM as the best scenario in the worst-best scaling.

**Table 2:** The associations (*N* = 1610) between the different decision-making styles and the intention to follow through with the vaccination/treatment recommendations (“expected adherence”)

	Expected adherence-%	SD	Mean rank	χ^2^ANOVA (3)	*p*	W
Vaccination						
Decision-making styles:						
*Authoritarian*	72.3	0.448	2.44	95.33	< 0.001	0.018
*Guiding*	73.4	0.442	2.46			
*Simple*	75.4	0.431	2.50			
*Shared*	80.1	0.400	2.60			
Treatment						
Decision-making styles:						
*Authoritarian*	62.2	0.485	2.52	165.77	< 0.001	0.032
*Guiding*	57.2	0.495	2.42			
*Simple*	57.0	0.495	2.41			
*Shared*	69.1	0.462	2.65			

Post hoc analysis of within-subject contrasts determined the effect sizes among the different styles of decision-making in the prospective treatment adherence: Among the scenarios of vaccination recommendation adherence, the most significant difference was between the shared and the authoritarian decision-making (W = 0.04). Among the scenarios of treatment recommendation adherence, SDM differed most significantly from scenarios of simple decision-making (W = 0.08) and guided decision-making (W = 0.07).

### Retrospective and prospective views of SDM associating with expected adherence

In the next stage of the analysis, expected adherence to vaccination and treatment was examined in multivariable models (Table 3). Adherence after SDM was set as an outcome variable that encompassed the prospective view of decision-making. The retrospective experience of SDM (SDM-Q-9 scale) was included in the model as an explanatory variable. The control variables were the respondent’s age, gender, subjective health, and frequency of healthcare service usage.

**Table 3:** Prospective adherence after shared decision-making associated with retrospective experience of shared decision-making (*N* = 629)

	Expected vaccination adherence in the scenario of shared decision-making				Expected treatment adherence in the scenario of shared decision-making			
	*B*	*OR*	*p*		*B*	*OR*	*p*	
Gender	0.337	1.401	0.166		0.253	1.288	0.196	
Age	-0.011	0.989	0.581		0.008	1.008	0.616	
Subjective health	-0.259	0.772	0.075		0.207	1.231	0.083	
Healthcare service use	0.013	1.013	0.951		-0.358	0.699	< 0.05	
Prior experience of shared decision-making	0.042	1.043	< 0.005		0.052	1.053	< 0.001	
Constant	1.965	7.132	0.220		-0.659	0.518	0.611	
Nagelkerke R^2^	0.04				0.06			

In the first model, SDM-Q-9 was used to explain the variance in the expected vaccination adherence among those who were motivated by SDM. A higher motivation for the respondents to involve themselves in decision-making was found to depend on their prior experiences with SDM in a clinical appointment. The retrospective experiences demonstrated a significant association with prospective views toward SDM. The respondents who had experiences of SDM, had a four-percent higher likelihood to be motivated by SDM. This association remained constant in models in which the age, gender, subjective health, and/or frequency of healthcare service usage were controlled.

In the second model, SDM-Q-9 was used to explain the variance in the expected treatment adherence among those who were motivated by SDM. A higher motivation for the respondents to involve themselves in decision-making was found to depend on their prior experiences with SDM in a clinical appointment. The preference for SDM was associated with the retrospective experiences of higher-level SDM and a lower frequency of healthcare service usage. The participants who had experiences of SDM, had a five-percent higher likelihood to be motivated by SDM. No interaction was found between the frequency of healthcare service usage and SDM.

In comparing the pseudo-R2 coefficients, we found that the model fit was stronger in the treatment scenario than in the vaccination scenario. Although this is partly explainable by the fact that the frequency of healthcare service usage was significant only in the treatment scenario, it is not entirely the case. The difference was observable already in base regression models in which SDM alone predicted the adherence with treatment (pseudo-R^2^ = 0.04) more strongly than adherence with vaccination (pseudo-R^2^ = 0.03).

## Discussion

The study combined prospective *preferences* of SDM with retrospective *experiences* of realized SDM in clinical encounters. As the first objective of the study, an experimental survey instrument measuring the prospective SDM was assessed regarding its associations with the more subjective self-report scale, SDM-Q-9. The positive correlation between the retrospective perceptions and prospective experiences of SDM supported our vignette-testing hypothesis (H1). Most importantly, the respondents correctly recognized the valence of the experimental conditions. These findings establish convergent validity to the use of experimental vignettes in studies of SDM in which the traditional and straightforward self-report scale is complemented by explicit survey items with a more ambiguous study design.

In line with the second set of hypotheses, both the retrospective and prospective views about SDM were found to be associated with the intention to follow through with the received medical recommendations. The expected adherence to vaccination (H2a) and treatment (H2b) was higher among respondents who had retrospective experiences of and prospective motivations in SDM. Thus, SDM is viewed as a preferable scenario when it comes to the ideal outcome of following through with medical recommendations. This is in line with previous studies in which a constructive dialogue between the patient and the practitioner has been found to support adherence to treatment [6, 7]. For example, qualitative research on doctor-patient interaction shows how, in recommending a hearing device, patient expresses less rejection if their opinions are acknowledged [58]. SDM consists of sharing knowledge and information, as well as building a co-operational, conversational ethos in the relationship between a patient and a practitioner. These are valuable means to get the patient onto the same page with the doctor and vice versa. SDM is viewed as having a positive impact on the patient’s quality of life and as a counterinfluence on failure demand and pseudo-compliance [36].

By producing information regarding the patients’ intentions to follow through with given recommendations, this study also contributes to discussions about the reduction of failure demand, an unfavorable condition in terms of both healthcare outcomes and resources, especially in aging societies. Failure demand is considered a negative outcome because, as a result, the patient may leave the appointment with a need to find additional answers and service paths. Pseudo-compliance is a negative outcome because the patient may leave the appointment seemingly satisfied with a decision regarding the treatment but then decide to act against the recommendations and what the practitioner perhaps perceived as a mutual understanding. Compared to previous findings [36], the frequency of pseudo-compliance was relatively high in the data of Finnish pensioners. Adherence to medication recommendation after the hypothetical scenarios emerged in 72–80% of the responses, while adherence to treatment recommendation emerged only in 57–69% (Table 2).

When making a decision on appropriate treatment in the hypothetical scenarios, the greatest differences in expected treatment adherence were between guided and simple decision-making and SDM. This may be explained by the existence of the different treatment options to choose from and the awareness of potential harms. In SDM, the harms and benefits are discussed, and the patient’s individual situation is taken into account. A guided or simple decision is made without further reflection, with the doctor or the patient making the decision. The latter could also be seen as a form of consumerism [18]. In this case, the patient chooses the treatment option they want; however, not all patients are prepared for this role. In this study, the age of the respondent contributed to their decision-making preference. Over the years, older peoples’ experiences with decision-making in clinical encounters have followed an authoritarian model [59] rendering it an expected preference.

Retrospective experiences and prospective preferences regarding SDM played a more significant role in expected treatment adherence that in expected vaccination adherence. Again, SDM regarding the treatment options is a more complex situation, whereas the decision-making regarding vaccination is that of the simpler choice of receiving or refusing it. The finding can also be interpreted as signaling extant vaccine resistance as opposed to general treatment resistance [60]. Furthermore, in the deliberation regarding vaccination, doctor-centered, authoritarian decision-making appeared as the worst scenario in the best-worst setting. This differed from the treatment scenario, in which the worst scenario, on average, was the situation in which the patients would be expected to have a more dominant role in making the decision.

In other words, authoritarian decision-making was not demonstrated to be the worst scenario in expected treatment adherence, as it was in expected adherence to vaccination. In a treatment-decision context, involving patient to the decision-making process can even arouse feelings of doubt toward the practitioner, who seems to be unwilling to act as the sole decision-maker in the situation [61]. The finding may also be interpreted as another sign of attitudinal resistance toward vaccines, resulting in a rejection of any attempts to mandate vaccination, while decisions regarding treatment, in general, provide a more flexible basis for conversation.

### Implications for practice

As for academic literature, the study contributes to the discussions of healthcare-related decision-making by introducing a perspective combining together different methodologies as well as experiences and motivations toward SDM in the same study design. Retrospective experiences have been dominating the literature which has left out the future-oriented motivational aspect of decision-making. To expand the knowledge of the patients’ expectations on SDM, further cross-validation studies using various types of written and illustrated scenarios are encouraged.

SDM can be facilitated with different kinds of digital decision-making applications by which patient-doctor interaction is promoted and information about treatment options, risks, and benefits can be shared [62]. Among older people living at home or in community care, these practical tools and aids may be essential in clarifying the patients’ views and values as important elements in health-related decision-making and patient empowerment [1, 63].

### Limitations

The results, including the lack of interaction effect between the frequency of healthcare service usage and SDM, suggest that the findings are generalizable to older adults in Finland independent of their health status or use of healthcare services. However, the low response rate may adversely impact the representativeness of the sample and, consequently, limit the generalizability of the study.”. The study was likely subjected to a selection bias due to the use of an electronic survey which limits the generalizability to those older adults who have motivation and the means to use computers or smart devices. Yet, in this case, it should be acknowledged that, regardless of the fact that the electronic data collection limited the respondents to those who have email addresses and an access to a computer or a smart device, the sample utilized represents more than 1.3% of the entire population of 120,000 pensioners.

While we observed a correlation between SDM and adherence to medical recommendations, its predictive utility is viewed as moderate. This suggests the presence of additional, more influential factors contributing to the multifaceted nature of adherence. As for the retrospective part of the questionnaire, SDM-Q-9 was one of the many possible scales available. The responses in SDM-Q-9 can be biased by the inaccurate recall of the past experiences. Memory recall errors were however minimized through a careful planning of the study design in which the six-week frame was found as an ideal one.

## Conclusion

This multi-method study combined the more conservative scale of SDM with an experimental vignette design in the attempt to produce more bias-robust knowledge on the importance of SDM in healthcare encounters. The retrospective and prospective perspective of SDM produced new knowledge on how SDM endorses older adults’ intentions to follow through with the treatment and vaccination recommendations.

The patient group of older adults is to be acknowledged as proactive healthcare consumers who, on average, gain from sharing information and decision-making. The findings show that the patients’ follow-through intentions of medical recommendations are supported by their experience of being involved in the decision-making process together with the practitioner. The multi-method study design shows promise in using vignettes to gain more subtle information about the interaction between patients and healthcare professionals.

### Electronic supplementary material

Below is the link to the electronic supplementary material.

Supplementary Material 1

Supplementary Material 2

## Data Availability

Data can be shared only after the project.

## Abbreviations

FPF: Finnish Pensioners’ Federation
SDM: Shared decision making
