# Supplementary material for: Shared decision-making endorses intention to follow through treatment or vaccination recommendations: a multi-method survey study among older adults
Source: BMC Med Inform Decis Mak. 2024 Jul 23;24:202. doi: 10.1186/s12911-024-02611-2 (PMC11264447; doi:10.1186/s12911-024-02611-2)
Supplement: Supplementary file 1 — Supplementary Material 1 [file 12911_2024_2611_MOESM1_ESM.docx]

APPENDIX A

You will be presented with two types of decision-making scenarios, both of which include four altering situations. The decision-making procedures are presented in cartoons and in bold text. The background information is identical in each of the four situations. Please relate to the role of the patient in the scenarios and respond to the scenario-related statements in the way in which you would in a similar situation.

Vaccination scenarios:

Background: You have a significant risk to catch a seasonal inflammatory disease for which there is a highly effective vaccination available. You are granted a free vaccination at your local health center. The inflammatory disease is generally a harmless one but, in rare cases, it can be fatal. The risk of getting the more serious type of the disease has not been associated with any chronic illnesses. Known adverse effects for the vaccination include soreness at injection site and fever, which are expected with some of the vaccinated. All adverse effects are not necessarily known. The vaccination gives a 90-percent protection against the inflammatory disease with a 12-month prevalence.

Situation 1: The doctor asks whether you would like to receive the vaccination, to which you give an affirmative response. How would you act in reality?


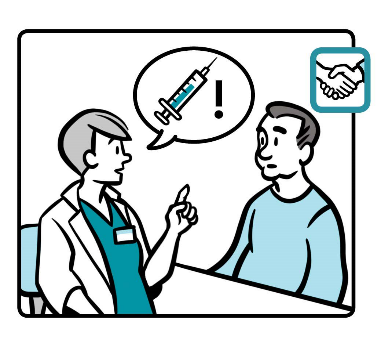


Situation 2: The doctor informs you about the efficacy and the adverse effects of the vaccination. The doctor states that it would be sensible to receive the vaccination, and this is decided. How would you act in reality?


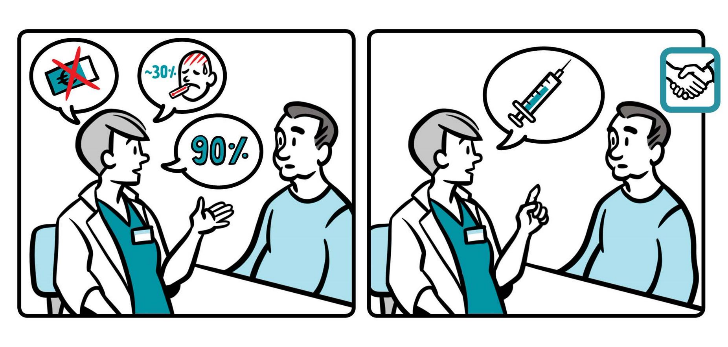


Situation 3: The doctor informs you about the efficacy and the adverse effects of the vaccination. The doctor then asks if you want to receive the vaccination. You give an affirmative response. How would you act in reality?


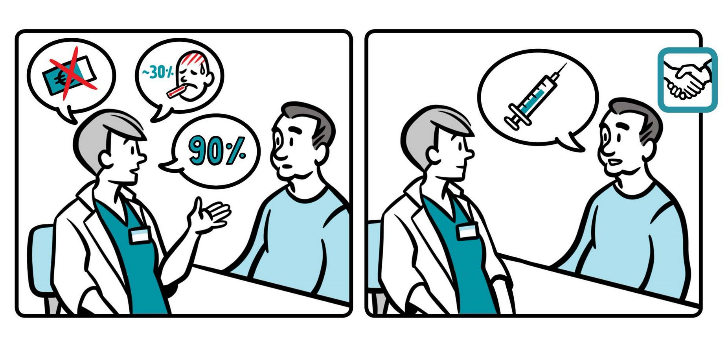


Situation 4: The doctor informs you about the efficacy and the adverse effects of the vaccination and asks for your opinion. You then discuss the positive and negative aspects concerning you, personally, taking the vaccination and decide together that it would be sensible to receive the vaccination. How would you act in reality?


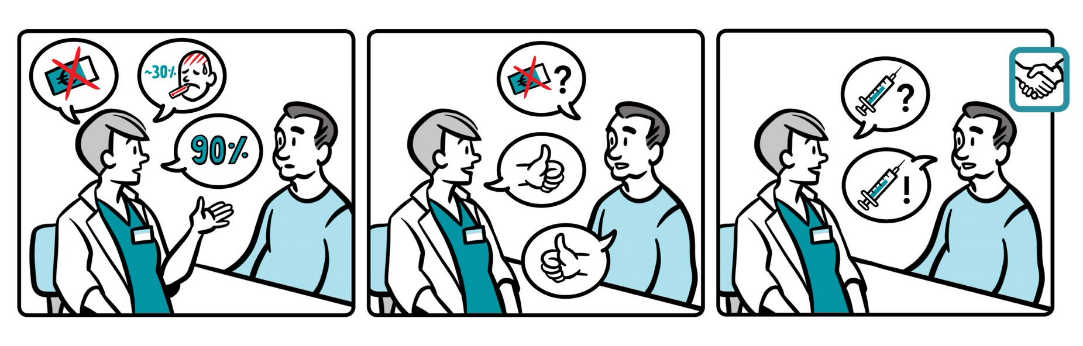


Response scale:

I will receive the vaccination without requiring any additional information.

Before getting the vaccination, I will look for additional information.

I will leave the appointment and pass the opportunity to take the vaccination.

Treatment scenarios:

Background: You have been diagnosed with a chronic illness for which there are different options for medication. The efficacies among the options are considered the same but the administration route, adverse effects, and the (safety) monitoring varies in them. The medication can be taken orally, as a medicated plaster, as a subcutaneous injection, or as an intravenous injection given periodically in a hospital.

Options:

- Monthly intravenous injections at a hospital: Adverse effects are extremely rare but serious, such as cancer or difficult inflammatory disease. Requires laboratory follow-ups every six months. The medication is delivered free of charge.
- Daily subcutaneous injections: Adverse effects are common but they are considered less serious, though they may affect the quality of life. The medication is delivered free of charge.
- Oral medication: Mild adverse effects for every user. Requires weekly laboratory follow-ups. The medication in delivered free of charge.
- Medicated plaster: Medication is highly tolerated without adverse effects. The need for laboratory follow-ups is marginal. Medication costs 600 euros per year.

Situation 1: The doctor introduces you to the most sensible option for medication and the decision is made accordingly. How would you act in this case?


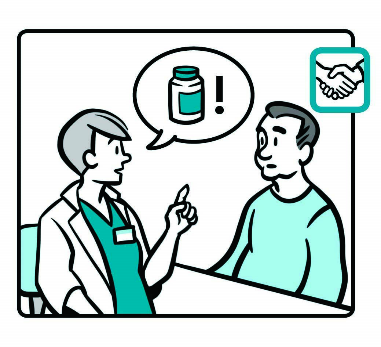


Situation 2: The doctor introduces you to the four options for medication with their individual pros and cons. The doctor then states which of the options would be most sensible in your situation and the decision is made accordingly. How would you act in this case?


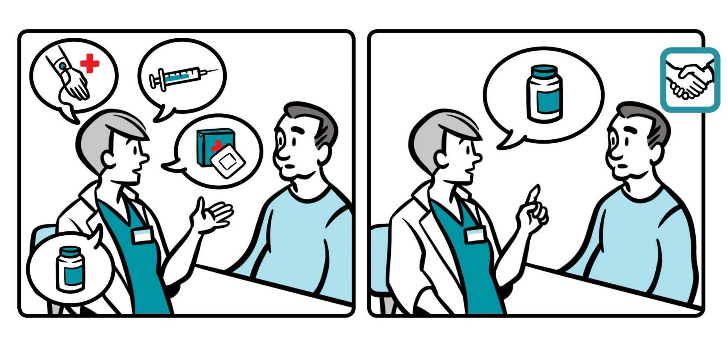


Situation 3: The doctor introduces you to the four options for medication with their individual pros and cons. The doctor then asks which one would be your choice. The decision is made accordingly. How would you act in this case?


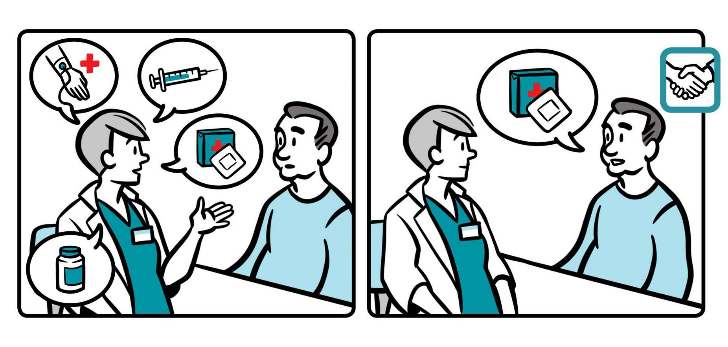


Situation 4: The doctor introduces you to the four options for medication with their individual pros and cons. The doctor then asks your opinion, you discuss the positive and negative aspects concerning your situation, and you decide together what would be the most sensible form of medication. How would you act in this case?


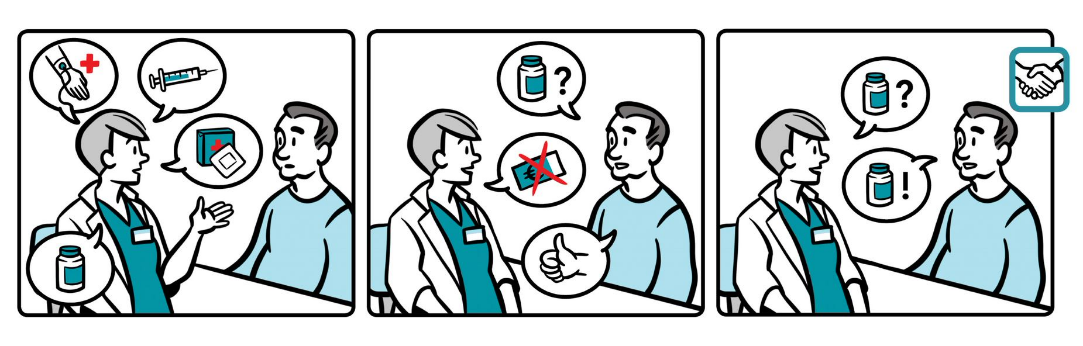


Response scale:

I will accept the treatment plan without requiring any additional information.

Before initiating the treatment, I will look for additional information.

I will leave the appointment with an intention not to initiate the treatment.
